# Design of the think PHRESH longitudinal cohort study: Neighborhood disadvantage, cognitive aging, and alzheimer’s disease risk in disinvested, black neighborhoods

**DOI:** 10.1186/s12889-023-15381-9

**Published:** 2023-04-03

**Authors:** Andrea L Rosso, Wendy M. Troxel, Tiffany L. Gary-Webb, Andrea M Weinstein, Meryl A. Butters, Alina Palimaru, Bonnie Ghosh-Dastidar, La’Vette Wagner, Alvin Nugroho, Gerald Hunter, Jennifer Parker, Tamara Dubowitz

**Affiliations:** 1 Department of Epidemiology, School of Public Health, University of Pittsburgh, Pittsburgh, US; 2 Division of Behavior and Policy Sciences, RAND Corporation, Santa Monica, US; 3 Department of Psychiatry, University of Pittsburgh, Pittsburgh, US; 4 Division of Economics, Sociology and Statistics, RAND Corporation, Santa Monica, US; 5 Survey Research Group, RAND Corporation, Santa Monica, US

**Keywords:** Alzheimer’s disease and related dementias, Lifecourse, Neighborhood, Racism, Mixed methods

## Abstract

**Background:**

Black Americans have disproportionately higher rates and earlier onset of Alzheimer’s disease and related dementias (ADRD) relative to White Americans. We currently lack a comprehensive understanding of how the lived experience and broader societal factors, including cumulative exposure to structural racism and the mechanisms underlying the risks, may contribute to elevated ADRD risk in Black Americans.

**Methods:**

The Think PHRESH study builds on existing, community-based research infrastructure, from the ongoing Pittsburgh Hill/Homewood Research on Neighborhood Change and Health (PHRESH) studies, to examine the contributions of dynamic neighborhood socioeconomic conditions across the lifecourse to cognitive outcomes in mid- and late-life adults living in two historically disinvested, predominantly Black communities (anticipated n = 1133). This longitudinal, mixed-methods study rests on the premise that neighborhood racial segregation and subsequent disinvestment contributes to poor cognitive outcomes via factors including (a) low access to educational opportunities and (b) high exposure to race- and socioeconomically-relevant stressors, such as discrimination, trauma, and adverse childhood events. In turn, these cumulative exposures foster psychological vigilance in residents, leading to cardiometabolic dysregulation and sleep disruption, which may mediate associations between neighborhood disadvantage and ADRD risk. This premise recognizes the importance of potential protective factors that may promote cognitive health, including neighborhood social cohesion, safety, and satisfaction. The proposed study will leverage our existing longitudinal data on risk/protective factors and biobehavioral mediators and will include: (1) up to three waves of cognitive assessments in participants ages 50 years + and one assessment in participants ages 35–49 years; clinical adjudication of ADRD will be completed in participants who are 50+, (2) extensive surveys of risk and protective factors, (3) two assessments of blood pressure and objectively measured sleep, (4) a comprehensive assessment of life and residential history; and (5) two rounds of in-depth qualitative interviews to reveal lifecourse opportunities and barriers experienced by Black Americans in achieving optimal cognitive health in late life.

**Discussion:**

Understanding how structural racism has influenced the lived experience of Black Americans, including dynamic changes in neighborhood conditions over time, is critical to inform multi-level intervention and policy efforts to reduce pervasive racial and socioeconomic disparities in ADRD.

## Background

Racial disparities in Alzheimer’s disease and related dementias (ADRD) are well-documented, with Black Americans having 2–4 times the risk of developing ADRD [1] and an earlier average age of onset compared to non-Hispanic Whites [2]. Such racial disparities have been largely attributed to differences in individual-level risk factors including medical comorbidities associated with ADRD, such as elevated cardiometabolic risk [3, 4]. Yet, research has clearly shown that systemic or neighborhood-level conditions underpin many individual-level risk factors in Black Americans [5–8].

**Fig. 1:**
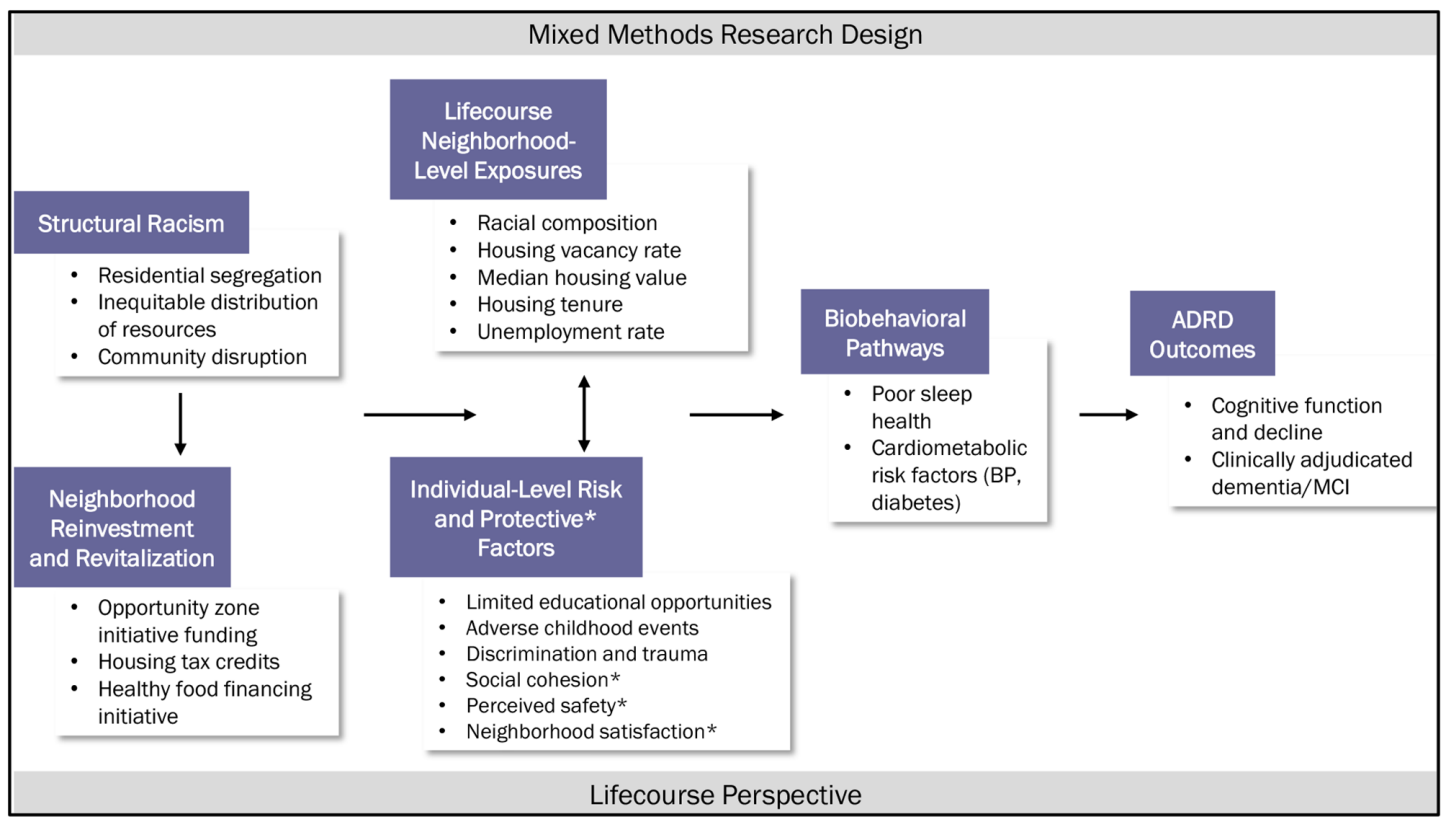
Conceptual framework of the impact of structural racism on neighborhood- and individual-level exposures across the lifecourse and pathways to cognitive function and risk for Alzheimer’s disease and related dementias

Structural racism reenforced by historic policies in the United States (US), including urban renewal projects, has contributed to segregated, disadvantaged, and disinvested Black communities across the US [9, 10]. Living in such neighborhoods is associated with lower cognitive function and less cognitive reserve [11–14] (Fig. 1). Higher cumulative exposure to stressors operating at the individual and neighborhood levels and lack of access to health-promoting resources are mechanisms that may contribute to greater cognitive decline and earlier onset of ADRD in Black Americans [4, 15, 16]. For example, repeated exposure to stressors associated with social and economic adversity and racial discrimination and marginalization may contribute to early deterioration across multiple body systems, including brain function [17–19]. Neighborhoods may be a particularly relevant source of these exposures as stronger associations between neighborhood disadvantage and cognitive function are observed in Black compared to White Americans [14, 20]. However, lifecourse patterns of exposure to racial and neighborhood stressors have not been systematically investigated despite the fact that variability in the types and timing of exposures (e.g., educational experiences [21] and stressful life events [22]) may differentially impact lifetime cognitive performance level compared with late-life cognitive decline.

The Think PHRESH study leverages rich, existing longitudinal data collected over more than a decade in a randomly sampled cohort of residents of two predominantly Black neighborhoods in Pittsburgh, PA. The parent study (Pittsburgh Hill/Homewood Research on Neighborhood Change and Health (PHRESH)) was initiated to identify causal relationships between changing neighborhood conditions and numerous health outcomes, including objectively measured diet, physical activity, sleep and cardiometabolic outcomes. By design, the two neighborhoods were matched on sociodemographic characteristics (Table 1). They have similar Area Deprivation Index scores with national percentiles of between 95 and 100, indicating severe economic deprivation [23]. Both neighborhoods are largely representative of other racially segregated and disinvested neighborhoods across the country. Yet, these two neighborhoods have experienced different trajectories and histories in terms of timing of exposure to various consequences of systemic racism and more recent commercial and economic development. While both neighborhoods suffered from substantial population loss between 1940 and present, differences in the timing of changes in neighborhood racial composition and socioeconomic conditions may impact cognitive outcomes differentially depending on timing of exposure within a participant’s lifetime and other lifecourse experiences.

**Table 1:** Characteristics of the two study neighborhoods from the 2014–2018 American Community Survey 5-year estimates

	% Black/African American	% Unemployed	% Below Poverty	% Female headed households	Median household income
Hill District	83%	17%	41%	29%	$24,421
Homewood	89%	18%	48%	29%	$20,336

The Think PHRESH study incorporates an explicit health equity focus and applies life-course and mixed-methods approaches. The aim is to clarify the impact of dynamic neighborhood conditions across the lifecourse and exposure to relevant racial and socioeconomic stressors and protective factors on cognitive function and ADRD risk in mid- and late-life Black Americans. In addition, key modifiable biobehavioral mediators including sleep and cardiometabolic health will be explored as more proximal intervention targets. The goal of this work is to substantially advance understanding of the impact of neighborhood factors on racial disparities in ADRD risk and open the door to systemic and multi-level intervention efforts.

## Methods/Design

### Study setting and sampling

The original PHRESH study began in 2011 as the “Pittsburgh Hill/Homewood Research on Eating, Shopping and Health” using a natural experiment design to evaluate the impact of opening a full-service supermarket on resident dietary behaviors and obesity in the Hill District neighborhood, compared with Homewood, both in Pittsburgh, Pa. Both neighborhoods were food deserts without access to fresh and healthy foods; one of the two, Hill District, gained a supermarket in 2013. Multiple additional research studies have been conducted, resulting in five waves of surveys through 2021. In the original PHRESH study design, homes in the neighborhoods were randomly selected and the primary food shopper of each home was asked to participate, resulting in a predominantly female sample. By design, the original sample consisted of two-thirds Hill District residents and one-third Homewood residents. The PHRESH projects have employed a community based participatory research approach which has included training neighborhood residents to be data collectors and employing a full-time field coordinator familiar with both neighborhoods; as a result, sample retention has been high with about 80% retention at each wave since 2011.

For Think PHRESH, we will invite currently enrolled PHRESH participants ages 35 years and older who had prior blood pressure and sleep data collected in 2016 or 2018 to participate starting in fall 2022. By including participants aged 35+, we will be able to capture mid-life cognitive performance in addition to late-life change, allowing us to explore age modification of our associations and sensitive exposure periods. With an anticipated retention of 80% of the sample (based on prior wave-to-wave retention rates), we project that we will enroll 833 current PHRESH participants during Wave 1 of the study. Existing cohort members will be enrolled through postcards and follow-up by telephone. Recruitment of an additional 300 participants is planned during Wave 1 for a total sample size of 1,133 participants. New recruitment will focus on increasing the share of Homewood residents with 200 new participants coming from that neighborhood and another 100 recruited from the Hill District. Using a full listing of residential addresses as our sampling frame, we will use a probability sampling approach paired with door-to-door recruitment to enroll a random sample of households. We have planned a multi-stage recruitment process: (1) postcards with study information will be sent to the randomly selected addresses to explain the study and invite households to call the field office to enroll; (2) for household addresses who did not call into the office, data collectors go to addresses and leave door hangers if no one is there; visiting up to 10 times.

### Community centered approach

A critical component of the sustainability and success of the PHRESH studies has been a commitment to strong community partnerships with organizations from both study neighborhoods. Throughout the study, there have been two Community Advisory Boards (one for each neighborhood) who have served as the voice of the community and who have provided feedback on study protocol, results, and revisions to the project plan. In addition, as part of our community-based research platform, our field coordinator (LW), who has been with the study since 2011, and study data collectors were hired from the communities. The field coordinator’s responsibilities include ongoing outreach to community partners and participants to enhance retention of the sample. In addition, we have worked with community organizations in both neighborhoods to help bolster awareness of the study and to disseminate findings periodically.

### Think PHRESH pilot

Prior to the Think PHRESH proposal, our team conducted an NIA-funded (R01CA149105-07S1; R01HL131531-03S1) pilot study from March 2019- February 2020 to assess willingness to participate and interest in a study focused on cognitive outcomes, as well as providing preliminary data on key study constructs. Existing PHRESH participants (n = 256) who were 50 years or older with at least one prior wave of complete data collection, including a blood draw and objective measurement of sleep, were enrolled in the Think PHRESH pilot study [24]. Of the participants who were contacted to participate in the pilot study, 93% consented to participate. Pilot testing included a complete neuropsychological assessment and clinical adjudication for cognitive impairment or dementia as described below.

### Study design

**Fig. 2:**
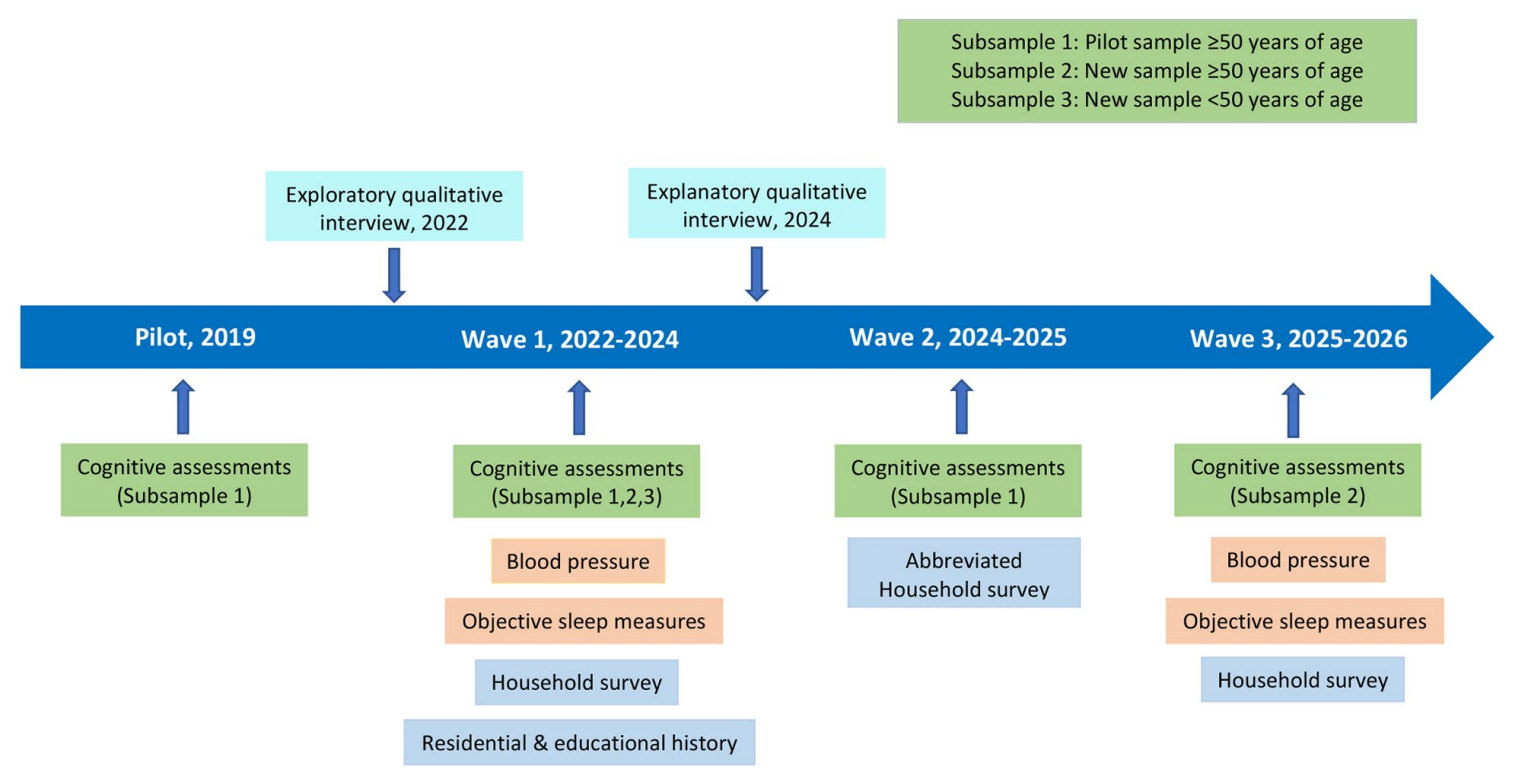
Study design and timeline for the Think PHRESH study

Think PHRESH is a mixed-methods study with up to 3 waves of quantitative surveys including a clinical neuropsychological assessment and 2 waves of qualitative interviews. The number of neuropsychological assessments each participant will receive depends on participant age and whether they were enrolled in the pilot sample. Participants are identified by 3 subsamples (Fig. 2): younger participants aged 35–49 will receive 1 neuropsychological assessment (wave 1), older participants (50+) who were enrolled in the pilot will receive two additional neuropsychological assessments (waves 1 and 2) for a total of three assessments, and older participants (50+) who were not in the pilot study will receive two neuropsychological assessments (waves 1 and 3). To minimize participant burden and fatigue associated with assessments, household surveys will be conducted separately from the neuropsychological assessments. All participants will be asked to complete all 3 waves of household survey data collection. The household surveys will be completed in-person during waves 1 and 3 to allow measurement of anthropometrics and blood pressure and to provide accelerometers for objective measurement of sleep. Wave 2 will be conducted by phone and will include a shorter household survey and administration of the Telephone Interview for Cognitive Status [25]. All in-person assessments (neuropsychological and household survey) will be conducted at the participant’s preferred location (e.g., their home, the PHRESH field office, or a neighborhood-based University of Pittsburgh Community Engagement Center). Data waves will be collected approximately every 18 months between fall 2022 and 2026.

### Qualitative interviews

Two rounds of qualitative interviews will be conducted both to help inform survey development as well as to inform interpretation of results (Fig. 2). Prior to the start of quantitative survey data collection, we recruited 60 individuals (n = 24 living in Homewood, n = 28 living in the Hill District, and n = 8 who used to live in Homewood or Hill but relocated elsewhere) from the current cohort of participants for exploratory in-depth interviews. This recruitment goal aligns with evidence suggesting samples between 20 and 30 are sufficient to ensure thematic and meaning saturation [26, 27]. Existing members of the PHRESH cohort were randomly sampled to participate in hour-long interviews conducted by phone which will be recorded and later transcribed verbatim. The questions explored perceptions of (1) lifecourse opportunities and barriers experienced by the respondents in relation to educational and residential histories; and (2) neighborhood conditions, such as residential stability, economic trends, and socio-cultural fabric, with a focus on historical changes. Through rapid analysis, these qualitative findings informed the subsequent quantitative data collection by assessing the importance of the survey domains to the participants, identifying potentially important domains that were not initially included, and generating hypotheses for testing.

The second round of qualitative interviews will be conducted after the completion of Wave 1 data collection. We will recruit 30 participants from the Think PHRESH sample who did not participate in the exploratory qualitative interviews to participate in one hour explanatory in-depth interviews. Questions will explore findings from initial analyses to help contextualize and explain results or mechanisms behind observed associations [28]. Questions will also probe strong but unexpected correlations, interactions, and unexpected non-significant findings and will be used in mixed methods analyses.

### Outcome assessment – cognitive function and impairment

#### Neuropsychological assessment

The battery of cognitive tests was chosen to assess general mental status and includes multiple measures within the language, visuospatial ability, attention, memory, and executive functions domains (Table 2). The battery is consistent with current conceptualizations of brain-behavior relationships and is congruent with many other robust studies of cognitive function. It is harmonized with several previous studies of older Black adults with similar demographic characteristics to provide appropriate norms and demonstrated reliability and validity [29]. These studies include the Hillsborough Elder African American Life Study [30], Chicago Health and Aging Project [31, 32], Heaton & Norman Demographic Correction Studies [33–35], Mayo’s Older African American Normative Studies [36], the Andreotti and Hawkins norms and the Health and Retirement Study [37]. The cognitive battery takes roughly 2–3 h and incorporates rigorous methodologies such as counterbalanced alternate forms.

**Table 2:** Think PHRESH neuropsychological battery and source of race-specific normative data

PHRESH Neuropsychological Assessment	Published norms	
Neuropsychological Test	Previous studies	Race-corrected norms in manual
**General Cognition/Literacy**		
Modified Mini-Mental State Exam (3MS)	X	
Wide Range Achievement Test (WRAT-5) Reading Subtest	X	X
**Language**		
15 Item Boston Naming Test	X	
Picture Naming (Repeatable Battery for the Assessment of Neuropsychological Status; RBANS)	X	
Controlled Oral Word Association Test- Letter Fluency	X	X
Category Fluency	X	X
**Visual-Spatial Ability**		
Consortium to Establish a Registry for Alzheimer’s Disease (CERAD) Constructional Praxis- Copy		X
Brief Visuospatial Memory Test-Revised (BVMT-R)- Copy		
**Attention**		
Digit Span (Wechsler Adult Intelligence Scale-IV (WAIS-IV))	X	X
Coding (WAIS-IV)	X	X
Trail Making Test Part A	X	X
Stroop Color Naming Condition	X	
Stroop Word Reading Condition	X	
**Memory Ability**		
Story Memory and Recall (RBANS)	X	
CERAD Word List Recall & Recognition	X	
BVMT-R Recall & Recognition		X
**Executive Functions**		
Trail Making Test Part B	X	X
Stroop Color-Word Interference Condition	X	
Clock Drawing Test		
**Diagnostic Criterion**		
ECog-38 Participant & Informant Questionnaire – complaints of cognitive decline		
PASS (Medication Management and Shopping subtasks) – Cognitive Instrumental Activities of Daily Living		

Additional data will include self-report of change in cognitive function [38], informant-reported change in cognitive function [38] when available, performance-based measures of cognitive instrumental activities of daily life (IADL) [39], medical history by the Charlson comorbidity index [40] along with additional dementia-relevant conditions such as history of traumatic brain injury and developmental learning disabilities, mental health disorders [41], and current medications.

#### Adjudication for cognitive impairment

For participants aged 50+ (projected N = 906), all data will be reviewed at consensus conferences to adjudicate cognitive classification and/or diagnosis. Consensus conferences will include a neuropsychologist, a geriatric psychiatrist, the neuropsychology examiner who completed the cognitive assessment, and the study coordinator. Participants aged 35–49 years will not have diagnoses adjudicated due to expected low base rates of ADRD. Data from the neuropsychological assessment will be used to adjudicate cognitive classification (i.e., normal cognitive function) and diagnoses (i.e., mild cognitive impairment (MCI) or dementia). We will apply the University of Pittsburgh Alzheimer’s Disease Research Center procedures [42] and use the NIA-AA criteria [43, 44] to adjudicate research diagnoses of MCI or dementia as appropriate. An NIA-AA diagnosis of MCI will be based on the following criteria: (1) concern regarding a change in cognition indicated by the participant or study examiner; (2) evidence of below expected cognitive performance in one or more cognitive domains, indicated by performance at -1 to -2 SD below expectation based on culturally appropriate norms on either two tests within the same cognitive domain or three tests scattered across cognitive domains; (3) relatively preserved functional independence, with at most mild difficulty on cognitively challenging IADLs. A diagnosis of dementia will be based on the same general criteria as above except that: (1) cognitive performance will be well below expectation, as indicated by performance at -2 or more SD below expectation and (2) evidence of functional dependence, with at least moderate difficulty on cognitively challenging IADLs. Participants who do not meet criteria for MCI or dementia will receive a classification of “normal cognitive status.” Adjudications will occur after all waves of cognitive assessments have been completed by a participant, to incorporate change data in diagnoses. Diagnoses will be assigned for all time points when cognitive assessment occurred.

### Risk factor assessment

Our data collection of risk factors for ADRD in this population is based on socio-ecological frameworks, lifecourse approaches, and an acknowledgement of the role of structural racism in the lives of Black Americans [45–47]. Domains covered are informed by these frameworks as well as results of our exploratory qualitative interviews described above. Risk factors are roughly grouped into four overarching domains here: childhood experiences, lifetime neighborhood characteristics, current risk and protective factors, and potential biobehavioral mechanisms.

#### Childhood experiences

Our data collection for childhood experiences focuses on educational opportunities, stressful adverse events, and housing experiences. These data will be collected only at Wave 1. Our education questions were adapted from the Health and Retirement Survey (HRS) Life History Questionnaire including questions that capture educational quality and individual academic performance [48–52]. Adverse childhood events [53, 54] will be captured by a standard questionnaire [55]. In addition, we will ask about participant birth location, parents’ birth locations, and parents’ educational attainment.

#### Lifetime neighborhood characteristics

Lifetime residential history will be captured at Wave 1 using methods adapted from HRS and the Reasons for Geographic and Racial Differences in Stroke Study (REGARDS). Up to 10 past residences and years living there will be captured via self-report [48–52]. In cases where exact addresses are not known, participants will be asked to provide landmarks, intersections, or any other information they are able to remember. At later Waves, residential history will be updated to capture moves since Wave 1. Prior work demonstrates reliability of residential history and suggests that long-term autobiographical data are preserved even in those with cognitive impairment [56–59]. These residential addresses will be geocoded and linked to historical census data available from 1940 to present. Census variables and their trajectories will allow us to capture exposure to urban decay, residential mobility, racial segregation, vacancy, and unemployment, which are shaped by structural factors such as urban renewal projects and subsequent neighborhood disinvestments. We hypothesize that these historical factors may contribute to cognitive outcomes by influencing educational opportunities, access to health-promoting resources, and exposure to stress.

Current perceived neighborhood conditions will be assessed by tapping into social cohesion [60, 61] and perceived safety [62, 63], and satisfaction with one’s neighborhood as a place to live [64]. Prior PHRESH studies have provided data on objective measures of neighborhood quality which have included audits of randomly sampled streets, greenspaces, and food retail venues. PHRESH has collected information on publicly funded investments in each neighborhood, and has used secondary data from administratively collected crime data, building permit data, and real estate sales data over time. These data have assisted with characterizing investment and change within the neighborhoods over time [65].

#### Current risk and protective factors

Risk and protective factors will be assessed at all Waves. Self-report of discrimination, psychological distress, and post-traumatic stress syndrome (PTSD) have been captured in PHRESH studies since 2016. Discrimination will be measured using the Everyday Discrimination Scale, Short Version [66]. PTSD will be measured using the validated 6-item PTSD Checklist (PCL-6) [67]. Psychological Distress will be measured using the Kessler-6 (K6), a validated assessment of general psychological distress [68]. Additional stressors that we will capture include perceptions of policing and loneliness [69]. Housing questions will cover lifetime experiences of housing insecurity including forced moves (evictions and foreclosures) [70] and problems with their current housing [71].

We will also capture protective factors including social support, psychological resilience, and religious coping. Social support will be captured as tangible support by the 4 item subscale of the Interpersonal Support Evaluation [72]. Resilience will be determined by the Brief Resilience Scale (BRS) [73] which assesses the ability to bounce back from stressful events. Religious coping will be reported by the positive religious coping subscale of the RCOPE which asks how participants use religion and/or a belief in a higher power to understand and deal with major problems in their lives [74].

#### Biobehavioral mechanisms

Three primary biobehavioral mechanisms will be assessed: objectively measured sleep and blood pressure, and self-reported cardiometabolic diseases. Blood pressure has been obtained in prior PHRESH waves (2016, 2018, 2021) and in the upcoming Think PHRESH Waves 1 and 3 using a A&D Medical automated blood pressure monitor after the participant is seated for five minutes. The average of two measurements will be used to calculate the average systolic and diastolic blood pressure. Self-reported cardiometabolic conditions have been collected regularly as part of PHRESH (2011, 2014, 2018, 2021) and will be reassessed at all waves of Think PHRESH as part of the clinical assessment. Participants will self-report chronic health conditions including diabetes, hypertension and heart disease, and medications for each.

Sleep has been collected at three prior waves of the PHRESH studies (2013, 2016, 2018) and will be collected as part of Think PHRESH at Waves 1 and 3. As in prior PHRESH data collection [75–79], participants will be asked to wear an ActiGraph (ActiGraph, LLC; Ft. Walton Beach, FL) GT9X accelerometer for 7 consecutive days and nights. Activity counts derived from the GT9X are an objective indication of the intensity of bodily movement and will be used to estimate sleep duration and efficiency (the percentage of time spent asleep/time in bed). Daily sleep diaries will be used to record bedtimes and wake-up times, to set the “rest” interval for actigraphy-derived sleep outcomes, and further verified by visual inspection of the actigraphy tracings. Sleep outcomes will be averaged across all available nights. Participants with fewer than four nights of actigraphy data will be excluded from analyses, consistent with recommendations for the minimum nights required to establish reliable sleep-wake patterns via actigraphy [80]. Processing protocols will follow the validated approach used in prior PHRESH studies [24, 81]. Specifically, actigraphy data will be scored using the GGIR R-Package which uses the raw accelerometer signal to identify sleep and wake periods. This scoring method has been validated against polysomnography with 83% ^82^ accuracy for identifying sleep and wake periods [82].

### Covariates

All covariates will be assessed at all waves and are available from prior years for all existing PHRESH participants. Individual-level current socioeconomic status will be operationalized with three indicators: educational attainment, annual household income, and employment status. We will collect home and automobile ownership as indicators of wealth. Race/ethnicity will be derived from two self-reported items, one on race and the other on ethnicity (i.e., Hispanic, non-Hispanic). Additional measures include age (date of birth), sex, marital/cohabitation status, and interviewer-measured body mass index (kg/m2).

### Study aims

Overall, our study aims are to examine how structural racism across the lifecourse has contributed to lived experiences of urban, low-income Black Americans that may underlie the heightened risk for ADRD in this population. We will achieve this aim through several planned analyses which will include analysis of the exploratory qualitative interviews to elucidate lifecourse opportunities and barriers experienced by Black Americans in achieving optimal cognitive health in late life. We will also quantify lifecourse exposure to neighborhood-level socioeconomic conditions (e.g., 1940-present change in racial composition) and their association with current cognitive outcomes. Further, we will examine the impact of cumulative exposure to race-relevant risk and protective factors measured at the individual level (e.g., discrimination, social support) and their interactions with neighborhood-level conditions (e.g., racial composition, social cohesion) on cognitive outcomes. Finally, we will determine how disruptions in biobehavioral mechanisms including objectively measured sleep and cardiometabolic risk (i.e., high blood pressure, diabetes) are related to cognitive outcomes in middle- and late-life Black Americans through a sequential mixed methods design.

### Ethical considerations

This protocol has been reviewed and approved by the Institutional Review Boards of both RAND Corporation and the University of Pittsburgh. All participants will provide informed consent to participate. Prior to obtaining consent, the data collector will go over the consent form and procedures with all potential participants. In order to assess capacity to consent, all participants will be asked “can you describe to me two of the things you will be doing for this study?” at the time of consent. Any individuals who are unable to answer this question will not be enrolled.

### Availability of data and materials

All data that the investigators will use in publications will be available to outside analysts to support replication and in concordance with NIH data sharing guidelines. Preparation for resource sharing will begin in year 1 of the project. We will prepare a data request form that will be available on our website (https://www.rand.org/well-being/community-health-and-environmental-policy/projects/phresh.html) or by contacting the study Principal Investigators (TD, WMT, ALR) via email.

## Discussion

The data generated by this longitudinal, mixed-methods study of cognitive function and the lifecourse will add unique and critical components to our understanding of how the lived experiences of Black Americans impacts the disproportionate risk of ADRD in this population. Notably, this study includes (1) longitudinal cognitive assessments, (2) in-depth qualitative interviews, (3) objective sleep and cardiometabolic health measures, (4) a comprehensive validated assessment of residential and educational history, and (5) clinical adjudications of ADRD diagnoses in participants who are 50 and older. The age distribution (range 35–95 years) and inclusion of life-history questions will allow for identification of early-, mid-, and late-life risk and resilience factors, and will provide a lifecourse perspective on how dynamic neighborhood conditions may differentially impact cognitive outcomes over time.

In summary, we will assess, both quantitatively and qualitatively, the impact of dynamic neighborhood conditions across the lifecourse and exposure to relevant racial and socioeconomic stressors and protective factors, as well as key modifiable biobehavioral mediators on cognitive function and ADRD risk in mid- and late-life Black Americans. The results of this work could substantially advance understanding of the impact of neighborhood factors on racial disparities in ADRD risk and may open the door to systemic and multi-level intervention efforts.

## Data Availability

All data that the investigators will use in publications will be available to outside analysts to support replication and in concordance with NIH data sharing guidelines. Preparation for resource sharing will begin in year 1 of the project. We will prepare a data request form that will be available on our website (https://www.rand.org/well-being/community-health-and-environmental-policy/projects/phresh.html) or by contacting the study Principal Investigators (TD, WMT, ALR) via email.

## List of Abbreviations

ADRD: Alzheimer’s disease and related dementias
PHRESH: Pittsburgh Hill/Homewood Research on Neighborhood Change and Health
IADL: Instrumental activities of daily life
MCI: Mild cognitive impairment
HRS: Health and Retirement Survey
PTSD: Post-traumatic stress syndrome
